# Supplementary material for: In silico characterization of bioactive phytochemicals as antivirals targeting the reovirus σ1 protein for inhibiting σ1-mediated host cell entry
Source: PLoS One. 2026 Jun 3;21(6):e0350009. doi: 10.1371/journal.pone.0350009 (PMC13232839; doi:10.1371/journal.pone.0350009)
Supplement: S1 File — (ZIP) [file pone.0350009.s001.zip › S1_file/Fig1.pptx]

## Slide 1
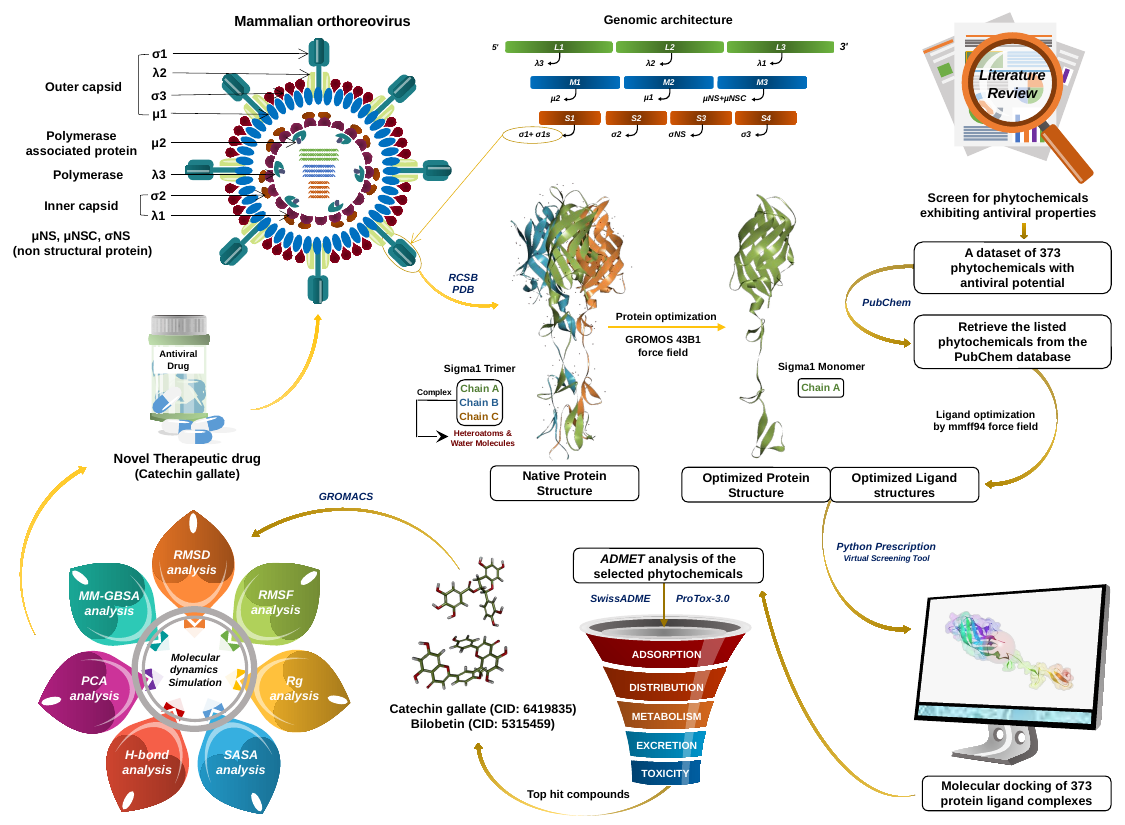

Mammalian orthoreovirus
Genomic architecture
Literature
Review
σ1
λ2
σ3
μ1
Outer capsid
μ2
Polymerase associated protein
Polymerase
λ3
σ2
Inner capsid
λ1
μNS, μNSC, σNS
(non structural protein)
5'
3'
L1
L2
L3
λ2
λ1
λ3
M1
M2
M3
µ1
µ2
µNS+µNSC
S1
S2
S3
S4
σ2
σ3
σ1+ σ1s
σNS
Screen for phytochemicals exhibiting antiviral properties
A dataset of 373 phytochemicals with antiviral potential
RCSB
PDB
PubChem
Protein optimization
GROMOS 43B1
 force field
Antiviral
Drug
Retrieve the listed phytochemicals from the PubChem database
Sigma1 Monomer
Chain A
Sigma1 Trimer
Chain A
Complex
Chain B
Chain C
Heteroatoms & Water Molecules
Ligand optimization by mmff94 force field
Novel Therapeutic drug
(Catechin gallate)
Native Protein Structure
Optimized Protein Structure
Optimized Ligand structures
GROMACS
RMSD
analysis
RMSF
analysis
MM-GBSA
analysis
Molecular
dynamics
Simulation
PCA
analysis
Rg
analysis
SASA
analysis
H-bond
analysis
Python Prescription
Virtual Screening Tool
ADMET analysis of the selected phytochemicals
SwissADME
ProTox-3.0
ADSORPTION
DISTRIBUTION
METABOLISM
EXCRETION
TOXICITY
Catechin gallate (CID: 6419835)
Bilobetin (CID: 5315459)
Molecular docking of 373 protein ligand complexes
Top hit compounds
