# Supplementary material for: In silico characterization of bioactive phytochemicals as antivirals targeting the reovirus σ1 protein for inhibiting σ1-mediated host cell entry
Source: PLoS One. 2026 Jun 3;21(6):e0350009. doi: 10.1371/journal.pone.0350009 (PMC13232839; doi:10.1371/journal.pone.0350009)
Supplement: S1 File — (ZIP) [file pone.0350009.s001.zip › S1_file/Fig2.pptx]

## Slide 1
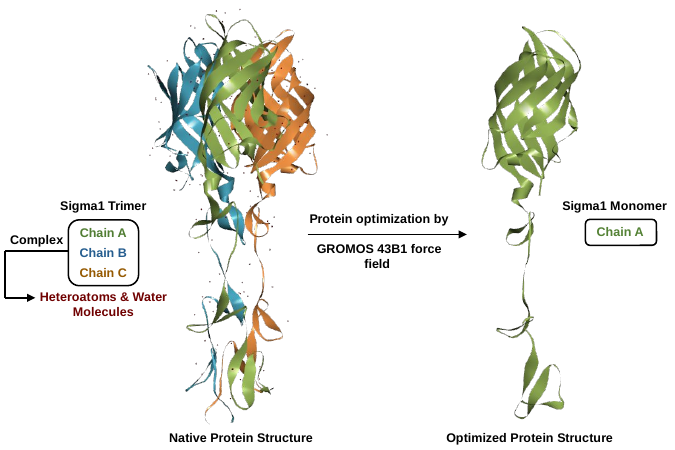

Sigma1 Trimer
Chain A
Chain B
Chain C
Complex
Heteroatoms & Water Molecules
Sigma1 Monomer
Chain A
Protein optimization by
GROMOS 43B1 force field
Native Protein Structure
Optimized Protein Structure
