# Supplementary material for: In silico characterization of bioactive phytochemicals as antivirals targeting the reovirus σ1 protein for inhibiting σ1-mediated host cell entry
Source: PLoS One. 2026 Jun 3;21(6):e0350009. doi: 10.1371/journal.pone.0350009 (PMC13232839; doi:10.1371/journal.pone.0350009)
Supplement: S1 File — (ZIP) [file pone.0350009.s001.zip › S1_file/Fig4.pptx]

## Slide 1
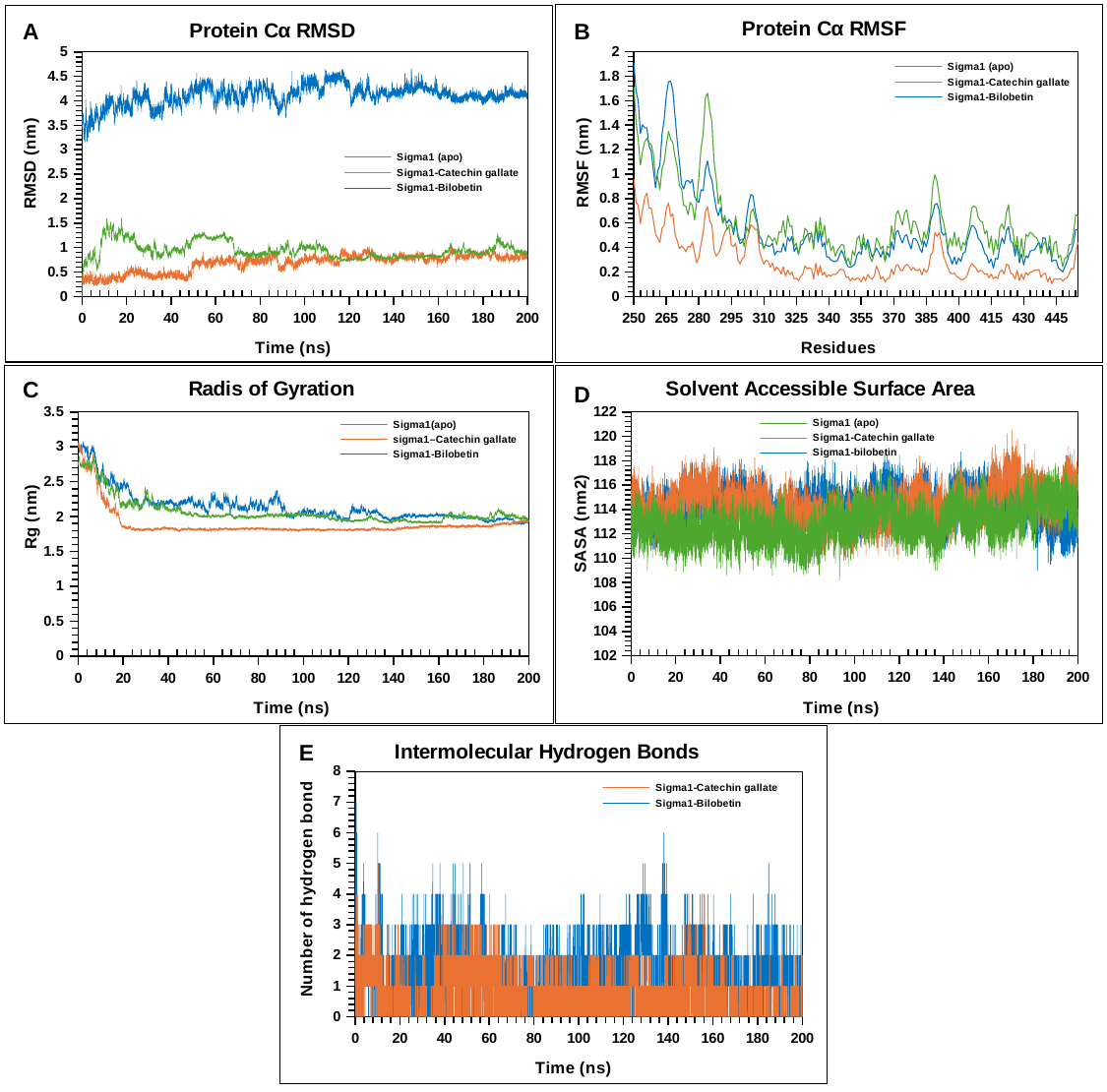

A
### Chart: Protein Cα RMSD
| Category | Sigma1 (apo) | Sigma1-Catechin gallate | Sigma1-Bilobetin |
|---|---|---|---|B
### Chart: Protein Cα RMSF
| Category | Sigma1 (apo) | Sigma1-Catechin gallate | Sigma1-Bilobetin |
|---|---|---|---|C
### Chart: Solvent Accessible Surface Area
| Category | Sigma1 (apo) | Sigma1-Catechin gallate | Sigma1-bilobetin |
|---|---|---|---|
### Chart: Radis of Gyration
| Category | Sigma1(apo) | sigma1–Catechin gallate | Sigma1-Bilobetin |
|---|---|---|---|D
### Chart: Intermolecular Hydrogen Bonds
| Category | Sigma1-Catechin gallate | Sigma1-Bilobetin |
|---|---|---|E
