# Supplementary material for: In silico characterization of bioactive phytochemicals as antivirals targeting the reovirus σ1 protein for inhibiting σ1-mediated host cell entry
Source: PLoS One. 2026 Jun 3;21(6):e0350009. doi: 10.1371/journal.pone.0350009 (PMC13232839; doi:10.1371/journal.pone.0350009)
Supplement: S1 File — (ZIP) [file pone.0350009.s001.zip › S1_file/Fig6.pptx]

## Slide 1
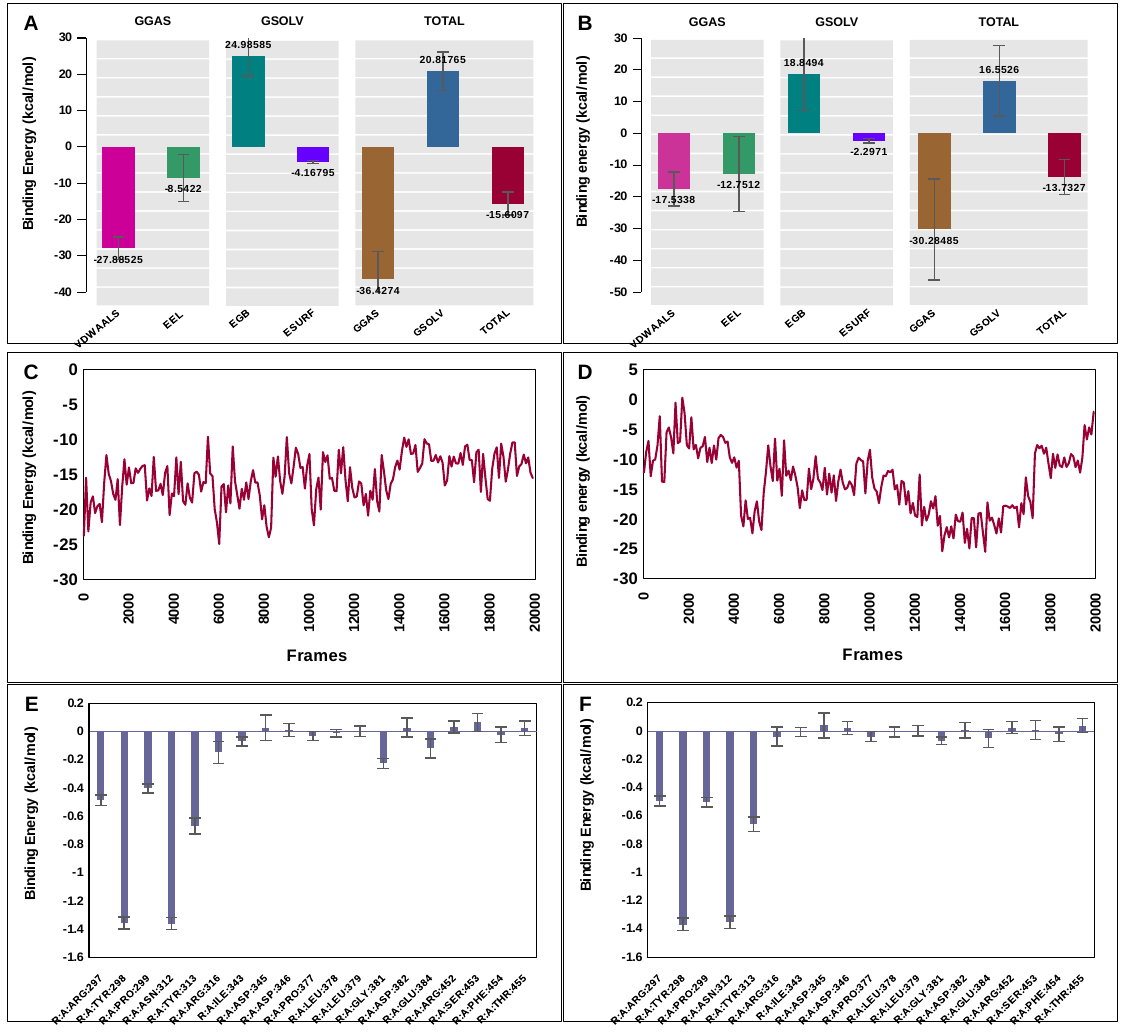

### Chart
| Category | |
|---|---|
| VDWAALS | -27.885249999999978 |
| EEL | -8.5422 |
| EGB | 24.98584999999998 |
| ESURF | -4.167950000000001 |
| GGAS | -36.42739999999998 |
| GSOLV | 20.817649999999993 |
| TOTAL | -15.609700000000005 |
### Chart
| Category | |
|---|---|
| VDWAALS | -17.533800000000003 |
| EEL | -12.75119999999999 |
| EGB | 18.849399999999992 |
| ESURF | -2.297100000000001 |
| GGAS | -30.284850000000006 |
| GSOLV | 16.552599999999995 |
| TOTAL | -13.732700000000005 |GGAS
GSOLV
TOTAL
GGAS
GSOLV
TOTAL
A
B
### Chart
| Category | |
|---|---|C
D
### Chart
| Category | |
|---|---|
### Chart
| Category | |
|---|---|
| R:A:ARG:297 | -0.485347476 |
| R:A:TYR:298 | -1.356678072 |
| R:A:PRO:299 | -0.401026416 |
| R:A:ASN:312 | -1.36080409199999 |
| R:A:TYR:313 | -0.667540612 |
| R:A:ARG:316 | -0.145329451999998 |
| R:A:ILE:343 | -0.0676695399999999 |
| R:A:ASP:345 | 0.0299240600000011 |
| R:A:ASP:346 | 0.0130897120000004 |
| R:A:PRO:377 | -0.0282242719999999 |
| R:A:LEU:378 | -0.0102437999999999 |
| R:A:LEU:379 | 0.00344999999999993 |
| R:A:GLY:381 | -0.223380263999999 |
| R:A:ASP:382 | 0.0302827479999987 |
| R:A:GLU:384 | -0.117629791999999 |
| R:A:ARG:452 | 0.0355487959999998 |
| R:A:SER:453 | 0.0676695399999999 |
| R:A:PHE:454 | -0.0203712719999997 |
| R:A:THR:455 | 0.0245831519999996 |E
### Chart
| Category | |
|---|---|
| R:A:ARG:297 | -0.495247476 |
| R:A:TYR:298 | -1.366738072 |
| R:A:PRO:299 | -0.501016416 |
| R:A:ASN:312 | -1.35060409199999 |
| R:A:TYR:313 | -0.657630612 |
| R:A:ARG:316 | -0.038769451999998 |
| R:A:ILE:343 | -0.00606953999999992 |
| R:A:ASP:345 | 0.0398240600000011 |
| R:A:ASP:346 | 0.0220897120000004 |
| R:A:PRO:377 | -0.0384242719999999 |
| R:A:LEU:378 | -0.00665999999999981 |
| R:A:LEU:379 | 0.00344999999999993 |
| R:A:GLY:381 | -0.0676695399999999 |
| R:A:ASP:382 | 0.00618026399999969 |
| R:A:GLU:384 | -0.0508827479999987 |
| R:A:ARG:452 | 0.0254487959999998 |
| R:A:SER:453 | 0.0070297919999999 |
| R:A:PHE:454 | -0.0223712719999997 |
| R:A:THR:455 | 0.0395831519999996 |F
