# Supplementary figures and images for: In silico characterization of bioactive phytochemicals as antivirals targeting the reovirus σ1 protein for inhibiting σ1-mediated host cell entry
Source: PLoS One. 2026 Jun 3;21(6):e0350009. doi: 10.1371/journal.pone.0350009 (PMC13232839; doi:10.1371/journal.pone.0350009)

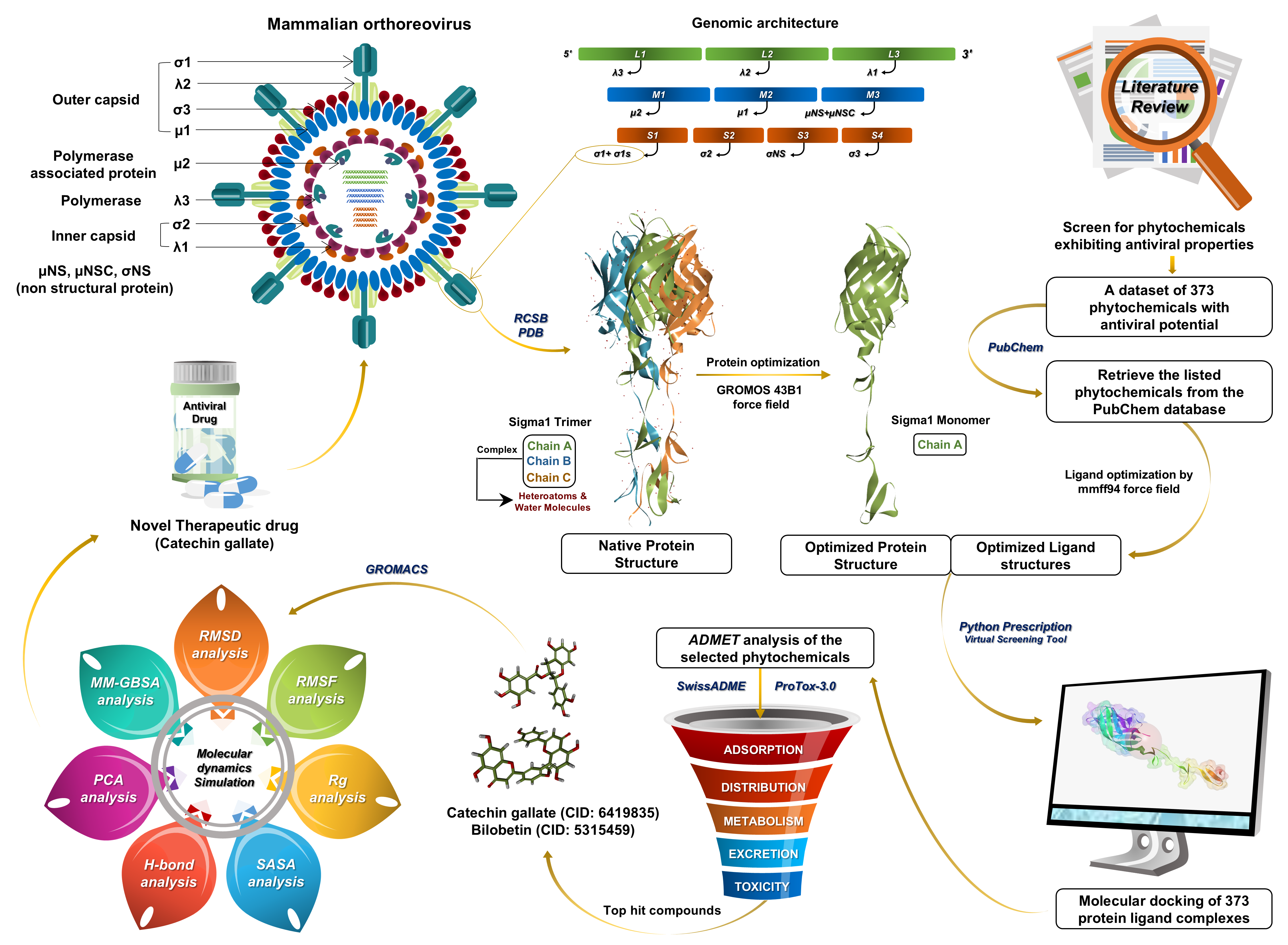

Supplement: S1 File — (ZIP) [file pone.0350009.s001.zip › S1_file/Fig1.tif]

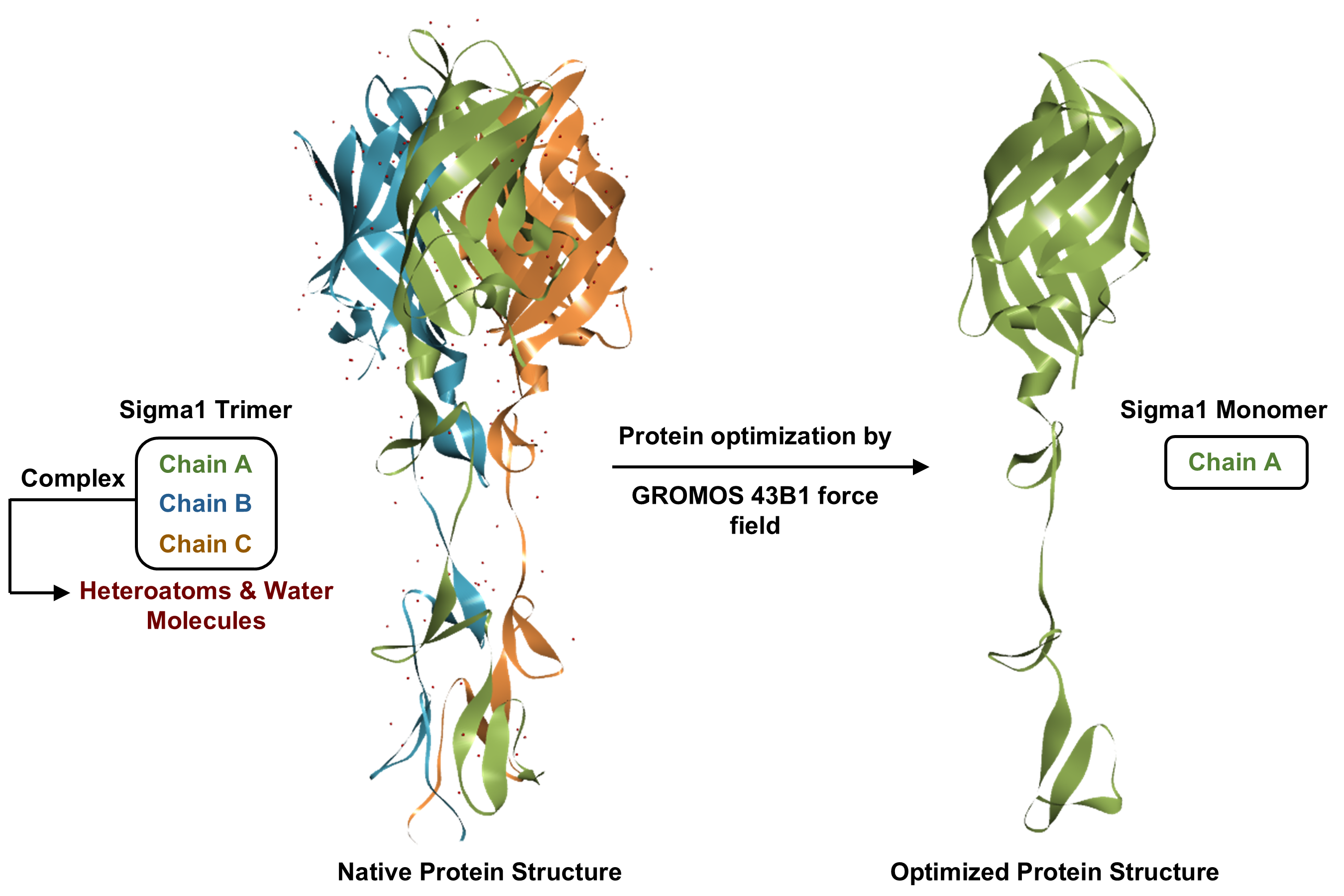

Supplement: S1 File — (ZIP) [file pone.0350009.s001.zip › S1_file/Fig2.tif]

## Slide 1
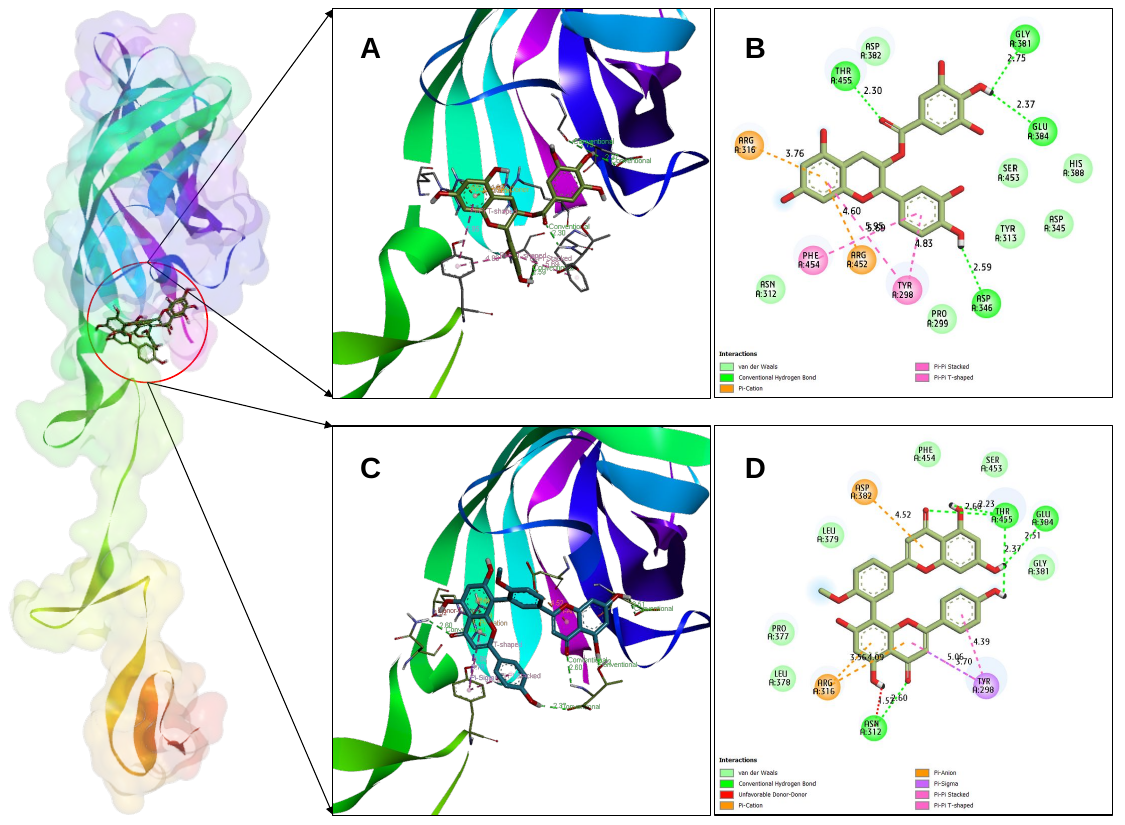

A
B
C
D

Supplement: S1 File — (ZIP) [file pone.0350009.s001.zip › S1_file/Fig3.pptx]

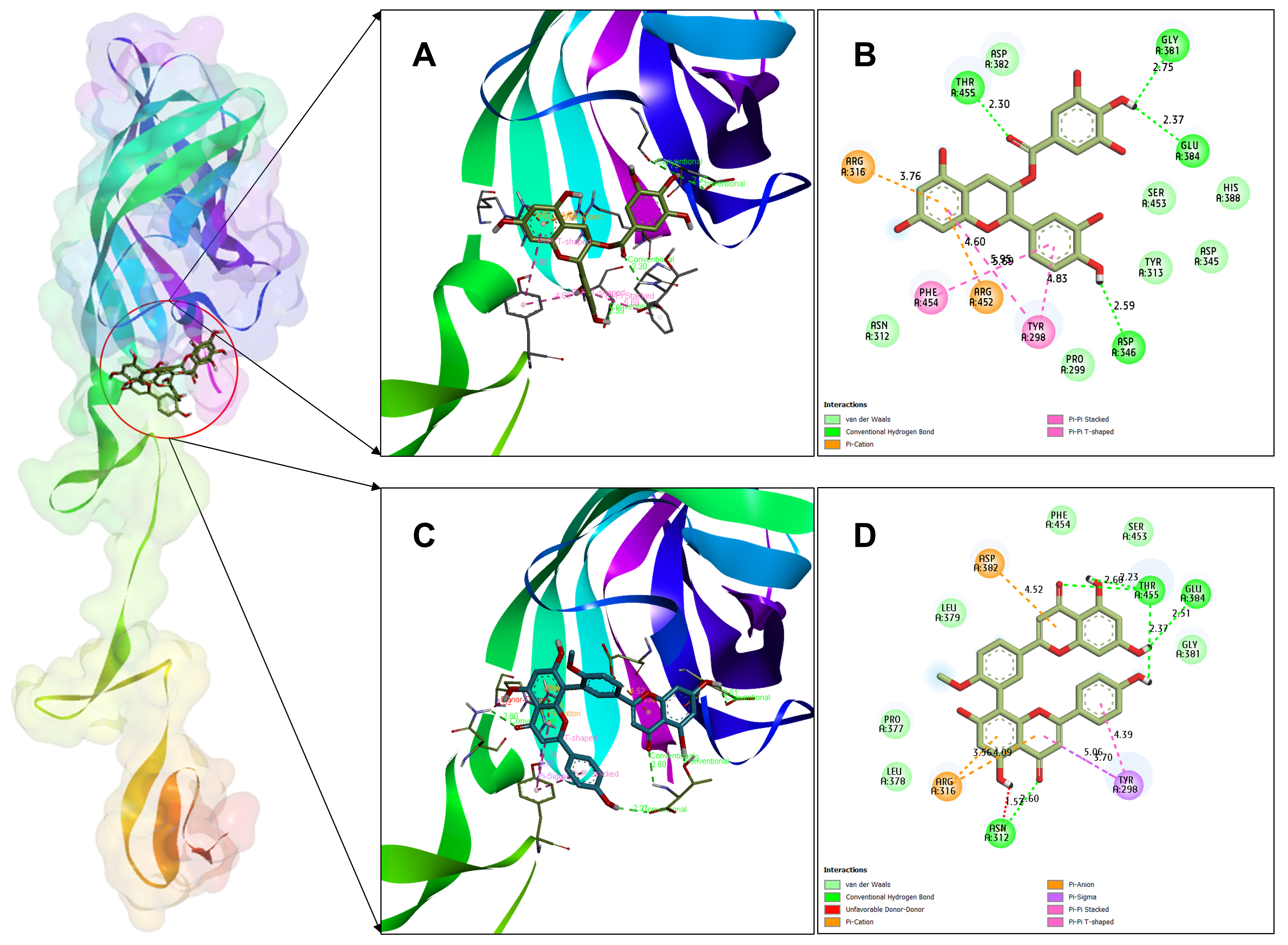

Supplement: S1 File — (ZIP) [file pone.0350009.s001.zip › S1_file/Fig3.tif]

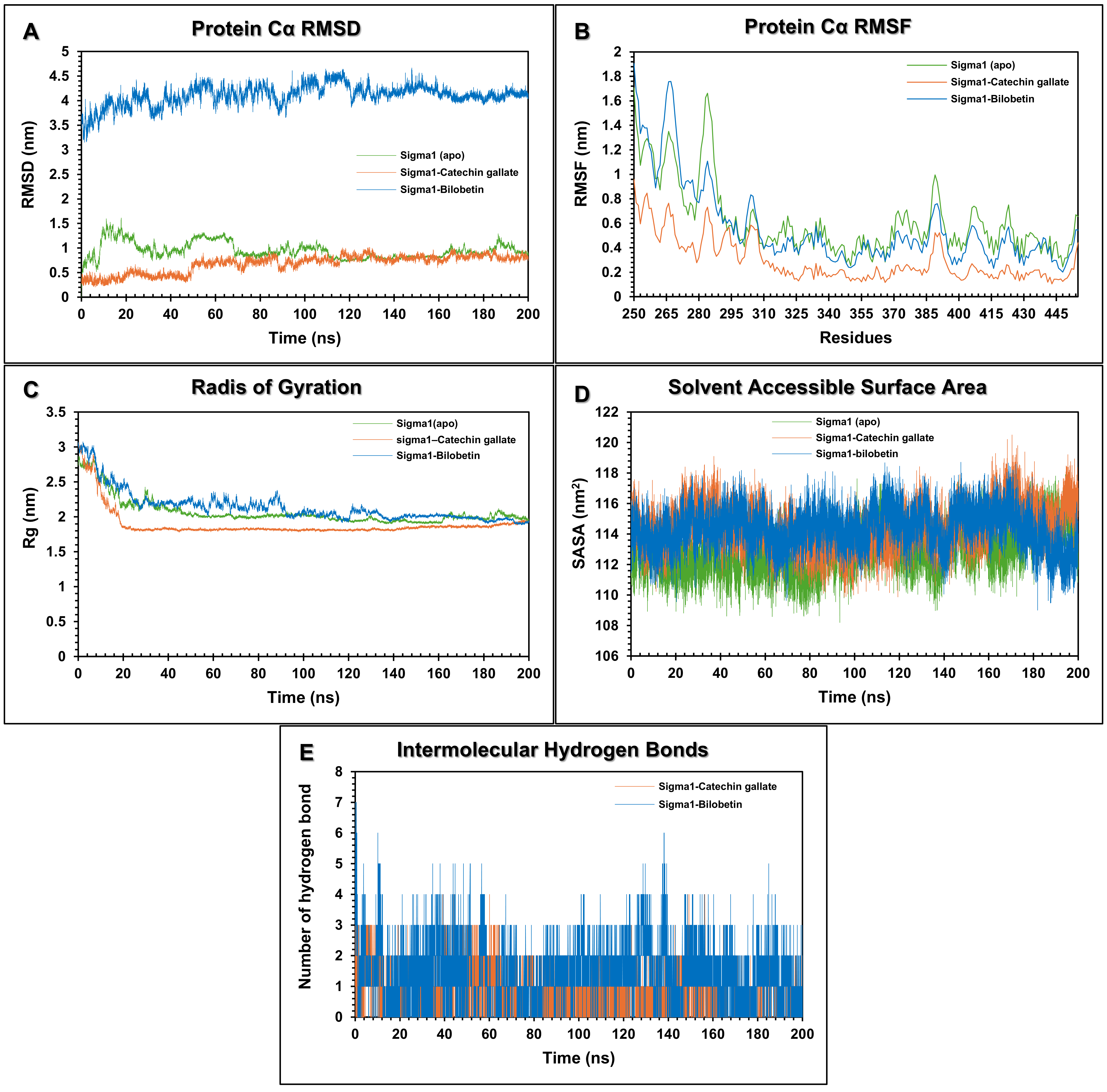

Supplement: S1 File — (ZIP) [file pone.0350009.s001.zip › S1_file/Fig4.tif]

## Slide 1
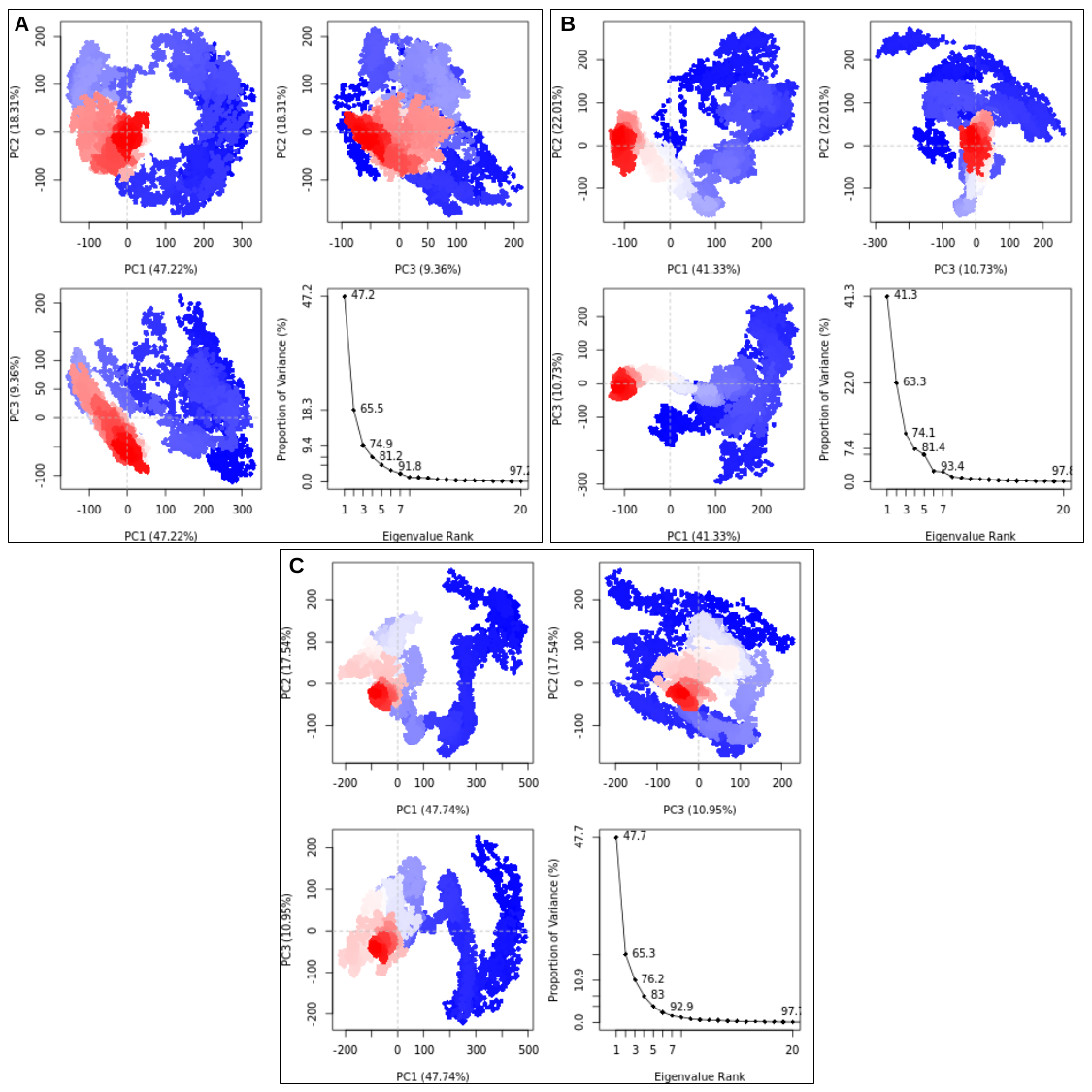

A
B
C

Supplement: S1 File — (ZIP) [file pone.0350009.s001.zip › S1_file/Fig5.pptx]

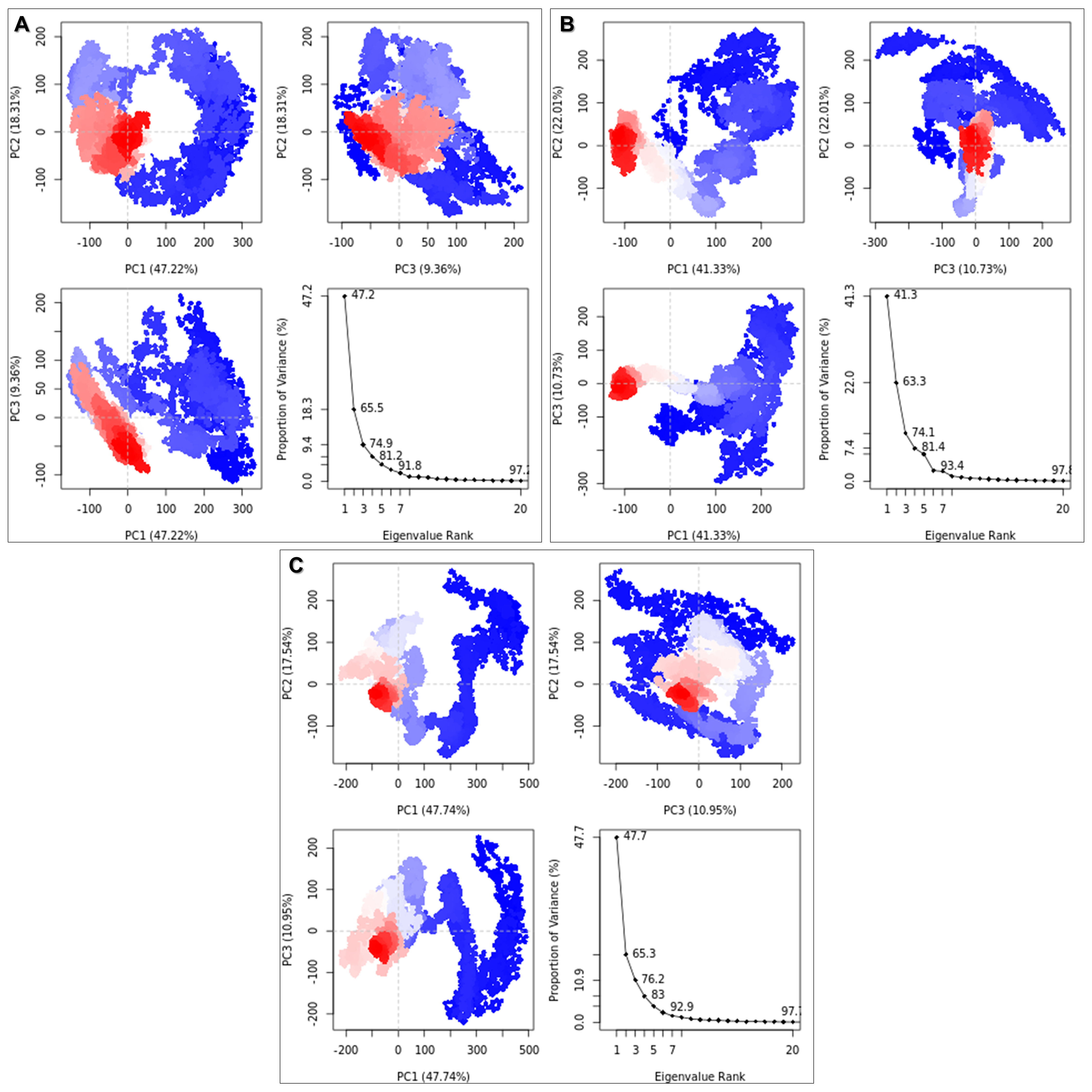

Supplement: S1 File — (ZIP) [file pone.0350009.s001.zip › S1_file/Fig5.tif]

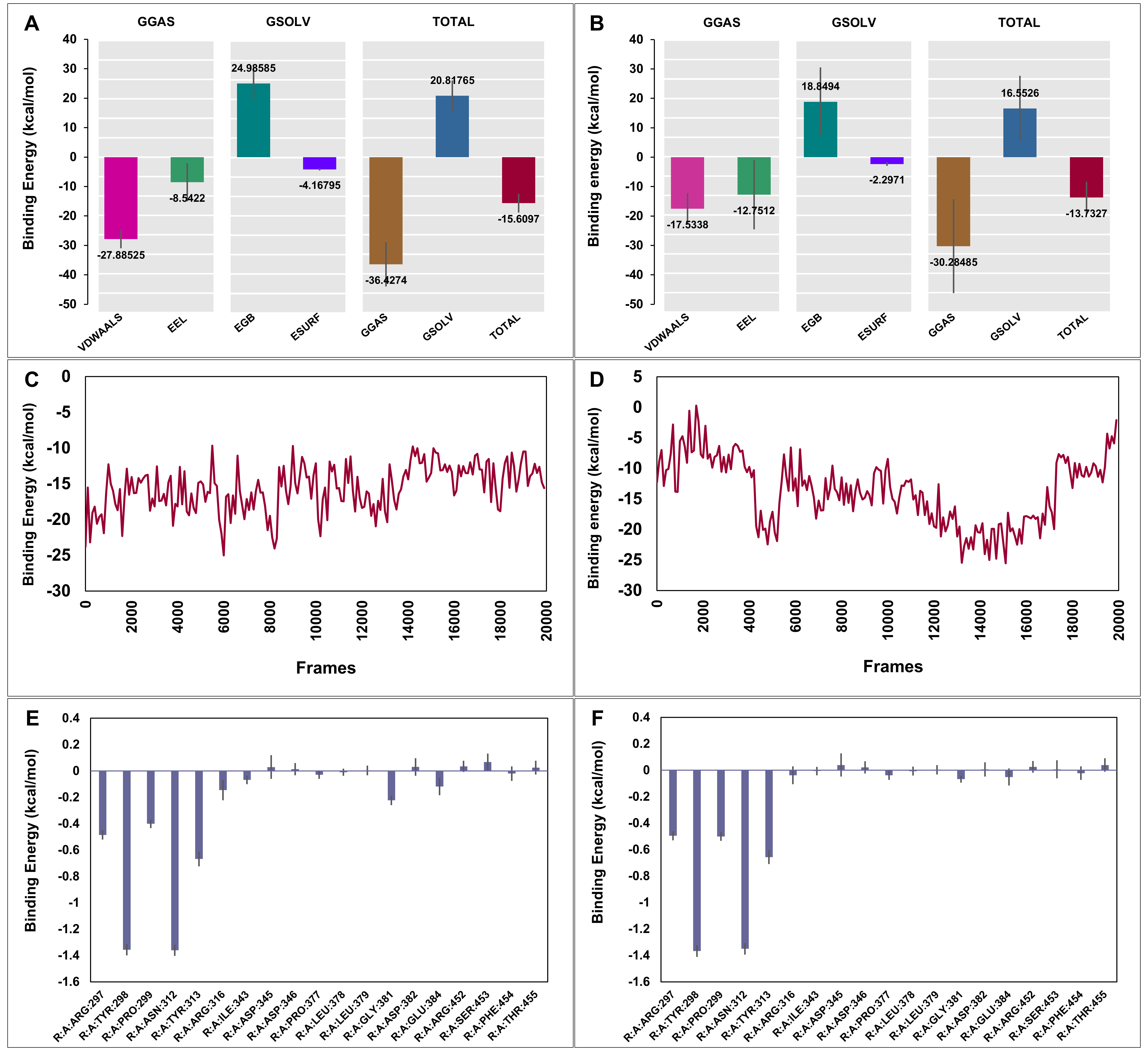

Supplement: S1 File — (ZIP) [file pone.0350009.s001.zip › S1_file/Fig6.tif]

## Slide 1
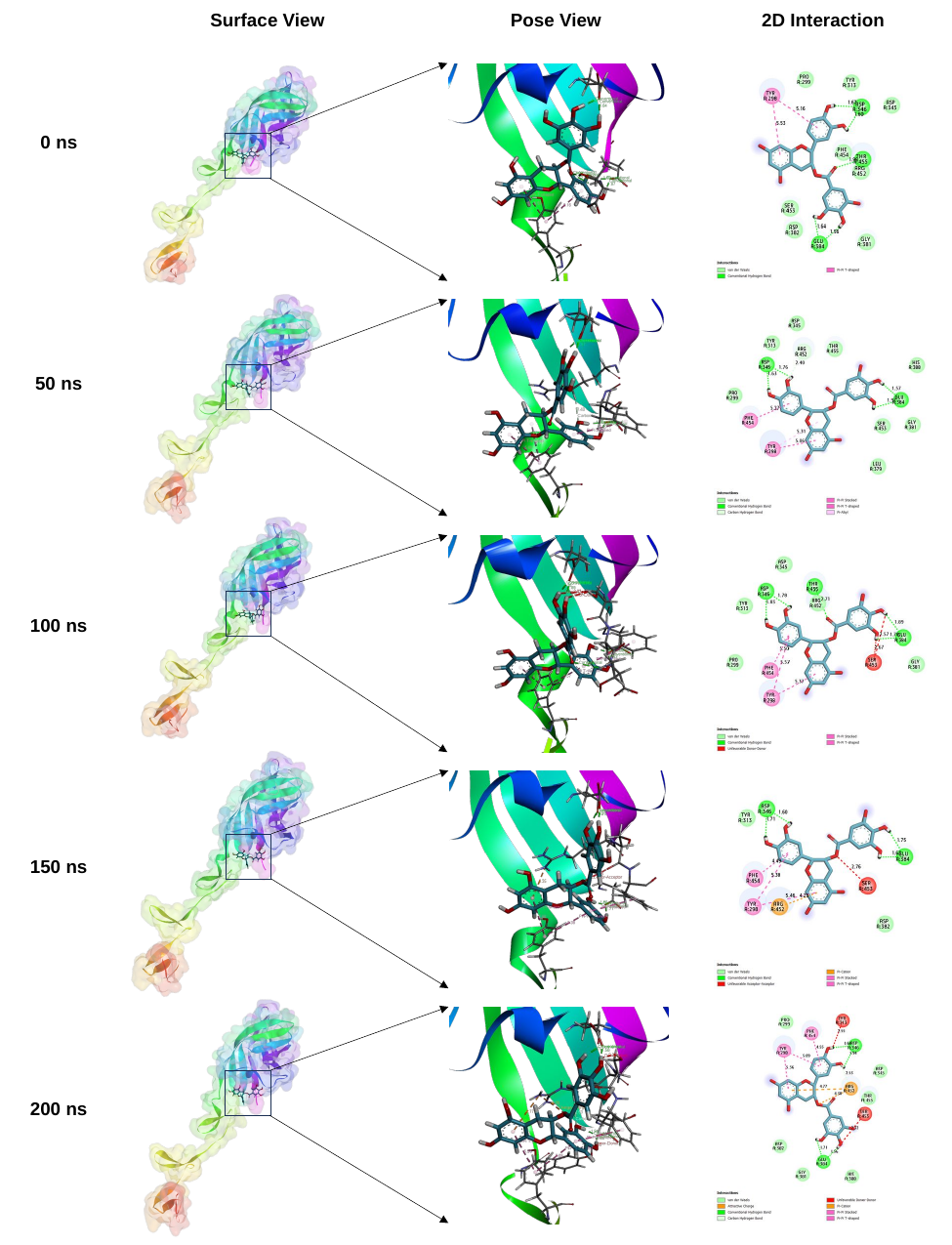

Surface View
Pose View
2D Interaction
0 ns
50 ns
100 ns
150 ns
200 ns

Supplement: S1 File — (ZIP) [file pone.0350009.s001.zip › S1_file/Fig7.pptx]

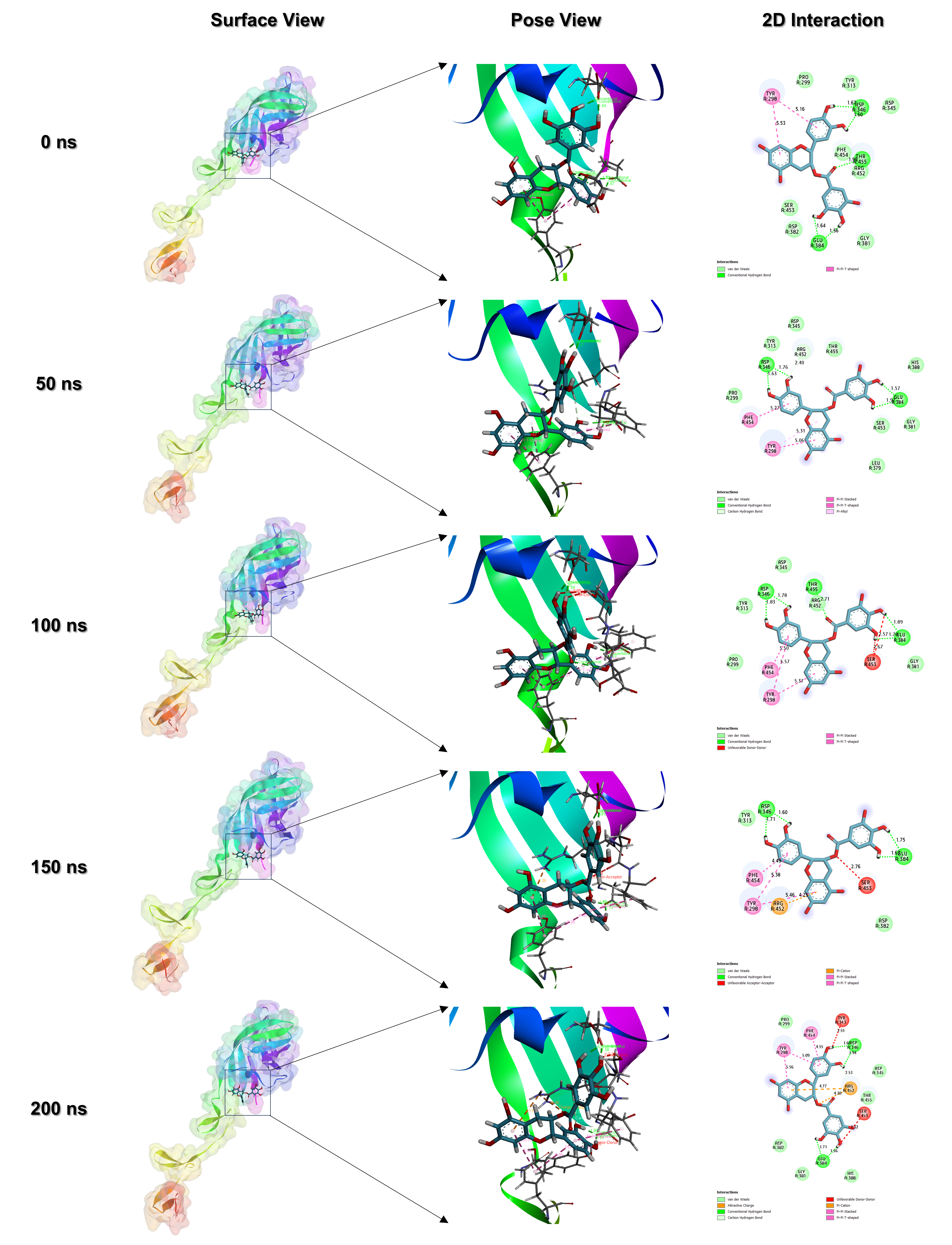

Supplement: S1 File — (ZIP) [file pone.0350009.s001.zip › S1_file/Fig7.tif]
