# Supplementary material for: In silico characterization of bioactive phytochemicals as antivirals targeting the reovirus σ1 protein for inhibiting σ1-mediated host cell entry
Source: PLoS One. 2026 Jun 3;21(6):e0350009. doi: 10.1371/journal.pone.0350009 (PMC13232839; doi:10.1371/journal.pone.0350009)
Supplement: Attachment — Submitted filename: Response to Reviewers.docx [file pone.0350009.s004.docx]

Date: April 15, 2026

Subject: **Manuscript Revision Notes**

Dear Dr. Sajjad Ahmad, Academic Editor, PLOS ONE,

We sincerely thank you for your email dated 27 March 2026 and for giving us the opportunity to revise our manuscript. We are also grateful to the reviewers for their careful evaluation and constructive comments, which have helped us improve the manuscript substantially.

In response to the reviewer comments, we have revised the manuscript thoroughly and addressed each point raised. All changes are marked in red in the revised manuscript, which has been uploaded as “revised manuscript with track changes.” Our detailed responses to the reviewers’ comments are provided below.

**Response to Reviewer 1**

We sincerely thank the reviewer for the thorough evaluation of our manuscript and for the constructive and insightful comments. We have carefully considered all points and revised the manuscript accordingly where possible. Our detailed, point-by-point responses are provided below.

**Major Comments**

**Comment 1:**

*Limited Novelty*

*Catechin gallate is a well-characterized flavonoid with well-proven antiviral and bioactive effects. The manuscript does not adequately illustrate how the present findings appreciably improve current understanding beyond verifying its general antiviral potential through docking experiments. The authors could properly contextualize the novelty of targeting σ1 specifically and highlight what new mechanistic insights are gained.*

**Response:**
We thank the reviewer for this important and insightful comment. We agree that catechin gallate is a well-known bioactive flavonoid with reported antiviral properties. However, the focus of our study was not to claim catechin gallate as a newly discovered antiviral compound, but rather to evaluate, within a curated set of previously reported antiviral phytochemicals, which compound showed the most favorable interaction with the σ1 attachment protein of reovirus.

To clarify the novelty of the present work, our study emphasizes the target-specific computational evaluation of σ1, which is the key viral attachment protein responsible for mediating host-cell recognition and entry. By focusing on σ1, we attempted to identify a plausible mechanism by which catechin gallate may interfere with the σ1-JAM-A interaction, thereby potentially disrupting viral attachment. Thus, the novelty of the study lies in the comparative screening of reported antiviral phytochemicals against a biologically relevant reoviral attachment target, rather than in the discovery of catechin gallate itself.

In the manuscript, we aimed to conceptualize the rationale for targeting σ1 as the principal viral attachment protein involved in host-cell recognition and entry. Specifically, in lines 60-70 and 98-111, we outlined the biological significance of σ1 and its role in mediating interaction with junctional adhesion molecule-A (JAM-A). These sections were intended to justify the selection of σ1 as a relevant antiviral target and to explain how interference with the σ1-JAM-A interaction may potentially disrupt viral attachment. Thus, the manuscript frames the study as a target-specific computational evaluation focused on the early stage of reovirus infection.

**Comment 2:**

*Inadequate Docking Verification*

*There is no validation for the docking protocol. The following is not reported in the manuscript:
• If a co-crystallized ligand is available, redocking it*

*• The application of a reference inhibitor*

*• Comparing to established σ1 binders.*

*The reliability of docking scores (−8.1 kcal/mol) is questionable in the absence of validation. The authors ought to offer comparative benchmarking or protocol validation.*

**Response:**
We thank the reviewer for raising this important methodological concern. We fully agree that docking validation is desirable for strengthening confidence in the results. However, in the present case, the available σ1 protein structure used in this study does not contain a co-crystallized ligand, and after an extensive literature search, we were unable to identify a reported reference inhibitor or any established small-molecule σ1 binder that could be used for redocking or benchmarking.

Because of this limitation in the available structural and experimental data, we were not able to perform the specific validation procedures requested by the reviewer. To avoid overstating the findings, we have clarified in the revised manuscript, specifically in the conclusion, that the docking results should be interpreted as computational predictions rather than experimentally validated binding data.

We appreciate the reviewer’s point, and we agree that future studies should incorporate experimental validation once suitable reference ligands or validated σ1 binder data become available.

**Comment 3:**

*Overinterpretation of Computational Findings*

*A number of claims suggest that viral attachment and translational potential are functionally inhibited. Claims indicating steric blockage of host-cell entry or therapeutic promise should be limited because this study is purely computational. It should be made very clear in the conclusions that these results are predictive and need to be confirmed by experiments.*

**Response:**
We thank the reviewer for this crucial suggestion. We have carefully revised the manuscript to remove or soften statements that may have implied direct functional inhibition or therapeutic confirmation. In the revised text, we now present the findings as predictive computational evidence supporting a possible inhibitory interaction, rather than as proof of antiviral efficacy.

We have also revised the Conclusion to clearly state that the results are hypothesis-generating and require experimental validation before any biological or therapeutic inference can be made. This clarification has been added on page 16, lines 603–607.

**Comment 4:**

*MM-GBSA and Sampling Concerns*

*Only 20 snapshots were used in the MM-GBSA analysis, which might not offer strong enough statistical confidence for a 200 ns trajectory. The authors ought to defend the choice of snapshots and think about boosting the sample density.*

**Response:**
We thank the reviewer for this valuable recommendation. We agree that using only 20 snapshots may limit the robustness of the MM-GBSA estimate for a 200 ns simulation. In response, we have increased the sampling density substantially in the revised manuscript and recalculated the MM-GBSA binding energy using 200 snapshots. We believe this expanded sampling provides a more reliable estimate of the binding free energy and improves the statistical confidence of the analysis.

This revision has been incorporated on page 11, line 392, and is also reflected in Figure 5.

**Minor Comments**

**Comment 1:**

*Clarify whether the σ1 structure used corresponds to a full trimeric form during docking or a monomeric chain.*

**Response:**
We thank the reviewer for this important clarification request. In the docking study, we used one chain (chain A) of the σ1 trimer rather than the full trimeric assembly. This choice was made because the σ1 chains are identical, and using a single chain was sufficient for the receptor-binding analysis while also reducing computational complexity.

We have now clarified this point in the Materials and Methods section under “Extraction and purification of the protein structure,” (line 183) and the selected chain is also shown in Figure 2.

**Comment 2:**

*Provide more detail on grid box justification for docking.*

**Response:**
We thank the reviewer for this important suggestion. The grid box was defined to cover the receptor-binding region of σ1 that mediates interaction with junctional adhesion molecule-A (JAM-A) during viral attachment. Based on the key residues reported in cited Article 70, we positioned the grid box to include the full interface implicated in host recognition, ensuring that the docking analysis focused on the biologically relevant binding site.

This justification has now been added on page 6, lines 223–224.

**Comment 3:**

*Include standard deviations for RMSD/RMSF where appropriate.*

**Response:**
We thank the reviewer for this helpful suggestion. In the revised manuscript, the RMSD and RMSF results now include standard deviation values where appropriate.

**Comment 4:**

*Improve figure clarity (especially PCA plots and MM-GBSA bar chart labels).*

**Response:**
We thank the reviewer for this useful comment. The figures have been revised to improve clarity, and the PCA plot and MM-GBSA bar chart have been relabeled to remove ambiguity. In the revised manuscript, Figures 5 and 6 correspond to the PCA and MM-GBSA analyses, respectively.

**Comment 5:**

*Consider comparing catechin gallate with structurally related flavonoids to strengthen screening relevance.*

**Response:**
We sincerely thank the reviewer for this excellent suggestion. We have strengthened the comparative aspect of the study by including bilobetin, the second-ranked compound from our docking-based screening, alongside catechin gallate. The revised manuscript now compares both compounds across the relevant computational analyses, which helps to better contextualize why catechin gallate emerged as the most favorable candidate against the reovirus σ1 protein.

**Response to Reviewer 2**

We sincerely thank the reviewer for the careful evaluation of our manuscript and for the constructive suggestions. We have addressed all comments thoroughly, and the manuscript has been revised accordingly. Our point-by-point responses are provided below.

**Comment 1:**

*The manuscript is written clearly, and sections are well planned by the authors.*

**Response:**
We thank the reviewer for this positive and encouraging comment. We are pleased that the clarity of the writing and the organization of the manuscript were appreciated.

**Comment 2:**

*There are some minor changes regarding some typos and grammatical errors. Make sure that the citation is properly cited and add some updated citations.*

**Response:**
We thank the reviewer for this valuable suggestion. The manuscript has been carefully revised to correct typographical and grammatical errors throughout. All references and in-text citations have been thoroughly checked for accuracy and consistency. In addition, recent and relevant literature has been incorporated where appropriate. Specifically, citations 9, 10, 11, 12, 14, 15, 18, 19, 21, 22, 28, 44, 46, 51, and 81 have been updated.

**Comment 3:**

*Abstract should be rewritten with incorporation of proper computational results.*

**Response:**
We thank the reviewer for this important suggestion. The Abstract has been revised to incorporate the key computational findings presented in the Results section, improving its accuracy and informativeness. However, highly detailed molecular dynamics parameters (e.g., RMSD, RMSF, Rg, SASA, and hydrogen bond profiles) were not explicitly included, as their proper interpretation requires contextual explanation. Including such details in the Abstract may reduce clarity and readability. We believe the revised Abstract achieves an appropriate balance between completeness and conciseness.

**Comment 4:**

*Introduction section is very short please make sure it has updated citations and some current investigation regarding the same topic portion must be revisited.*

**Response:**
We thank the reviewer for this helpful suggestion. The Introduction has been revised and strengthened with updated and relevant references. Specifically, citations 9, 10, 11, 12, 14, 15, 18, 19, 21, 22, 28, 44, 46, and 51 have been updated. We also conducted an additional literature survey to identify recent advances related to this topic; however, no substantially new findings were identified that warranted major expansion beyond the revisions already made.

**Comment 5:**

*Figure resolutions are not in a proper readable format; make sure it is easy to understand*.

**Response:**
We thank the reviewer for this comment. All figures have been carefully revised and replaced with high-resolution versions to improve readability and visual clarity. The updated figures are now clearer and easier to interpret.

**Comment 6:**

*Incorporate the pdb id in the methodology section as well with proper citation of the published articles.*

**Response:**
We thank the reviewer for this important suggestion. The PDB ID of the protein used in this study has been incorporated into the Materials and Methods section under the subsection “Extraction and purification of the protein structure.” The corresponding published article has also been properly cited. This information is provided on page 5, line 179 (Reference 60).

**Comment 7:**

*Proper scientific format for species names is required throughout the journal.*

**Response:**
We thank the reviewer for pointing this out. The manuscript has been carefully revised to ensure that all species names are presented in the correct scientific format throughout.

**Comment 8:**

*Do comparative investigation with the binding energies along the PCA analysis.*

**Response:**
We thank the reviewer for this valuable suggestion. A comparative analysis integrating binding energy results with principal component analysis (PCA) has been added to better correlate the thermodynamic stability with the dynamic behavior of the complexes. This addition can be found on page 15, lines 564–574.

**Comment 9:**

*What residues were mostly involved in the binding energies?*

**Response:**
We thank the reviewer for this important clarification. The revised manuscript now includes information on the key residues contributing to binding energy. Additionally, graphical representations illustrating residue-wise energy decomposition have been included (Figures 5E and 5F). These details are provided on page 12, lines 414–415.
