# Supplementary material for: In silico characterization of bioactive phytochemicals as antivirals targeting the reovirus σ1 protein for inhibiting σ1-mediated host cell entry
Source: PLoS One. 2026 Jun 3;21(6):e0350009. doi: 10.1371/journal.pone.0350009 (PMC13232839; doi:10.1371/journal.pone.0350009)
Supplement: Attachment — Submitted filename: Reviewer comments.docx [file pone.0350009.s002.docx]

**Reviewer comments**

The authors present a very interesting manuscript titled “**In-silico characterization of bioactive phytochemicals as antivirals targeting the Reovirus σ1 protein for inhibiting σ1-mediated host cell entry”**

” The presented study results are very interesting.

1 The manuscript is written clearly, and sections are well planned by the authors.
2 There are some minor changes regarding some typos and grammatical errors. Make sure that the citation is properly cited and add some updated citations.
3 Abstract should be rewritten with incorporation of proper computational results.
4 Introduction section is very short please make sure it has updated citations and some current investigation regarding the same topic portion must be revisited.
5 Figure resolutions are not in a proper readable format, make sure it is easy to understand.
6 Incorporate the pdb id in the methodology section as well with proper citation of the published articles.
7 Proper scientific format for species names is required throughout the journal.
8 Do comparative investigation with the binding energies along the PCA analysis
9 What residues were mostly involved in the binding energies?
